# Critical appraisal of the influence of antithyroglobulin on the measurement of thyroglobulin and thyroglobulin recovery

**DOI:** 10.1210/jendso/bvag183

**Published:** 2026-08-12

**Authors:** Robert Markewitz, Klaus-Peter Wandinger, Ralf Junker

**Affiliations:** Institute of Clinical Chemistry, University Hospital Schleswig-Holstein, Kiel 24105, Germany; Institute of Clinical Chemistry, University Hospital Schleswig-Holstein, Kiel 24105, Germany; Institute of Clinical Chemistry, University Hospital Schleswig-Holstein, Kiel 24105, Germany

**Keywords:** thyroglobulin, antithyroglobulin, thyroglobulin recovery, assay interference

## Abstract

**Background:**

Antibodies against thyroglobulin (anti-Tg) are known to be able to cause interference in the measurement of thyroglobulin (Tg). One method to quantify this interference is to measure Tg recovery after adding a known amount of Tg to the sample. The objective of this study was to examine the association between Tg, anti-Tg, and Tg recovery via a big data approach in real-life data.

**Methods:**

Results for Tg, anti-Tg and Tg recovery, measured in a high-throughput clinical laboratory via immunometric assays, were retroactively anonymized and analyzed. Specifically, the associations between Tg, anti-Tg, and Tg recovery with one another and with the variables age and sex were statistically evaluated.

**Results:**

15 609 samples were examined, of which only 98 samples exhibited disturbed Tg recovery. Of 66 of these, for which it was measured, 57 contained quantifiable and 28 elevated levels of anti-Tg. Anti-Tg was associated with decreased levels of Tg recovery and Tg itself (both *P* s< .0001), the strongest decreases being associated with elevated levels of anti-Tg. But even for elevated anti-Tg, median Tg recovery was well within the reference range at 94 ± 10.4%. Effect sizes of all statistical comparisons were small to very small.

**Conclusion:**

Tg recovery appears neither precise enough to detect interference of anti-Tg in the measurement of Tg on a single-specimen basis nor specific for it. This interference can nevertheless be detected statistically both for Tg recovery and for Tg, albeit with small effect sizes. Methods other than Tg recovery are needed to reliably detect disturbances in Tg measurement.

Thyroglobulin (Tg), a glycoprotein synthesized by the follicular cells of the thyroid gland as a precursor and storage form of T4 and T3 and released into serum, is an important marker for the presence of thyroid tissue (eg, in differentiated thyroid cancer [DTC]) (1). Antithyroglobulin antibodies (anti-Tg), present in as many as 10% of the general population (2) and 20% to 30% of patients with thyroid cancer (3-6), is a known challenge to the measurement of Tg as it can bind circulating free Tg within the serum, leading to spuriously low levels of Tg in immunometric assays (4, 7, 8). There is evidence that even very low levels of anti-Tg can cause interference in the measurement of Tg, and there is no apparent anti-Tg threshold value for this interference (3). As a possible solution, many manufacturers issue so-called Tg recovery assays. For these, a known quantity of Tg is added to the serum, and a repeated measurement of Tg is performed. If 70% to 130% (80-120% in other sources) are detected, no interference of anti-Tg in the measurement of Tg is assumed. However, there is still debate concerning the reliability of the Tg recovery assays (1). While the presence of anti-Tg does apparently not always cause interferences detectable by Tg recovery assays (3, 9, 10), in some cases, even small concentrations of anti-Tg can cause strong interferences (3, 4, 10). In 2019, a study showed very little benefit of performing a so-called mini-recovery test for Tg in DTC patients (11). However, these results cannot necessarily be generalized to other, more common Tg recovery assays and may not necessarily apply to non-DTC patients.

In the current study, we wanted to examine the association between Tg, anti-Tg, and Tg recovery using real-life data measured via immunometric assays in a high-throughput clinical laboratory. Purposefully, we did not limit our dataset to samples from patients with specific diagnoses, such as DTC, as we wanted to examine the real-life application of Tg and Tg recovery assays. Specifically, we set out to investigate how common the detection of increased levels of anti-Tg or of decreased levels of Tg recovery are and what influence they exert on each other's measurement and on the measurement of Tg itself. To this end, we collected a sample of all measurements of Tg (with Tg recovery) as well as all combined measurements of Tg, anti-Tg, and Tg recovery ordered within a period of more than 4 years in our laboratory for further statistical analysis. We hypothesized that elevated levels of anti-Tg would cause Tg recovery to be decreased and therefore that anti-Tg would be associated with lower measured levels of Tg. Furthermore, we expected that even nonelevated (quantifiable) levels of anti-Tg would exert a similar impact on the measurement of Tg recovery and Tg itself.

## Methods

### Study population

As a first step, all measurements of Tg (always combined with the measurement of Tg recovery in our laboratory) ordered at the Institute of Clinical Chemistry as part of the University Hospital Schleswig-Holstein (Kiel/Lübeck, Germany) within the period between June 9, 2016, and March 29, 2021, were retrospectively retrieved from the electronic archives. For the examination of the association between Tg, anti-Tg, and Tg recovery, all results from combined measurements of Tg, anti-Tg, and Tg recovery were retrieved as a subset of this larger cohort. The data were then deidentified and analyzed. Adopting a big data approach, there were no formalized considerations of sample size, and the data were not filtered for ordering department, which includes departments of nuclear medicine.

The study was approvingly acknowledged but ruled exempt from institutional board review by the Institutional Review Board (Ethikkommission) of the University of Lübeck (Germany).

### Assays

All 3 analytes (Tg, anti-Tg, and Tg recovery) were measured using the respective immunometric assays by Roche Diagnostics (Rotkreuz, Switzerland), specifically Elecsys Tg II, Elecsys Anti-Tg, and Elecsys Tg II Confirmatory Test, performed on the e801 module of the cobas 8000 (Roche Diagnostics, Rotkreuz, Switzerland) according to the manufacturer's instructions. Of note, in our laboratory and within the specified timeframe, all samples from which Tg was measured automatically also underwent Tg recovery testing. For Tg recovery testing with the Elecsys Tg II Confirmatory Test, 50 µL of reagent, containing a concentration of approximately 350 µg/L (with small, batch-specific fluctuations), was added to 200 µL of the undiluted sample. The limits of quantification, according to the manufacturer, are for Tg, 0.04 to 500 µg/L, and for anti-Tg, 10 to 4000 kIU/L. Results for Tg are reported in µg/L, results for anti-Tg in kIU/L, and results for Tg recovery in percentage of the added quantity of Tg. Results outside the range of measurement were included in the dataset and assigned a value 1 below or above the respective limit of quantification that was breached (eg, 0.039 µg/L for samples that returned a Tg result of <0.04 µg/L). There are no limits of quantification for Tg recovery, and the manufacturer defines a range of 70% to 130% within which the Tg recovery is assumed to be undisturbed by anti-Tg, while results outside this range lead to the assumption of the corresponding Tg result as disturbed. Furthermore, according to the manufacturer, the reference ranges for Tg and anti-Tg are 3.5 to 77 µg/L and <115 kIU/L, respectively.

### Statistical analysis

Prior to further analysis, normality of the distribution of the data was tested using the Shapiro-Wilk test, and normality was rejected for all 4 continuous variables: age, Tg, anti-Tg, and Tg recovery. Therefore, we performed nonparametric testing for all further analyses, using the Mann-Whitney *U* test for comparisons between 2 groups and Spearman's rank correlation for associations between 2 continuous variables. Effect sizes for Mann-Whitney *U* tests were calculated for statistically significant differences via the ρ statistic. For any test that examines effects on continuous variables, only quantifiable values were included in the analysis. Distributions of categorically scaled variables between groups were assessed using Pearson's χ² test, and associations found via this test were quantified via Cramer's V. Statistical significance was assumed for *P* < .05 and a statistical trend was defined as *P* < .10. Wherever average values with measures of dispersion are reported, these represent the medians with the median absolute deviation. All statistical analyses were performed using the open-source software for statistical computing and graphics R (version 4.1.0) with the integrated development environment RStudio (Version 1.4.1717) (12).

## Results

### Study population

Between June 9, 2016, and March 29, 2021, Tg (and therefore also Tg recovery) was measured in 15 609 separate samples from 14 338 individual patients. Of this set of samples, the combined measurement of Tg, anti-Tg, and Tg recovery was performed in 9824 samples from 8871 individual patients. Of these, 6214 patients (70.0%) were female. The median age of these patients was 56.9 ± 18.3 years. A Mann-Whitney *U* test revealed that the male patients, from whom samples were collected, were on average older than the female ones (60.4 ± 16.9 vs 55.5 ± 18.8; *P* < .0001).

### Descriptive statistics, associations with age and sex

**Figure 1:**
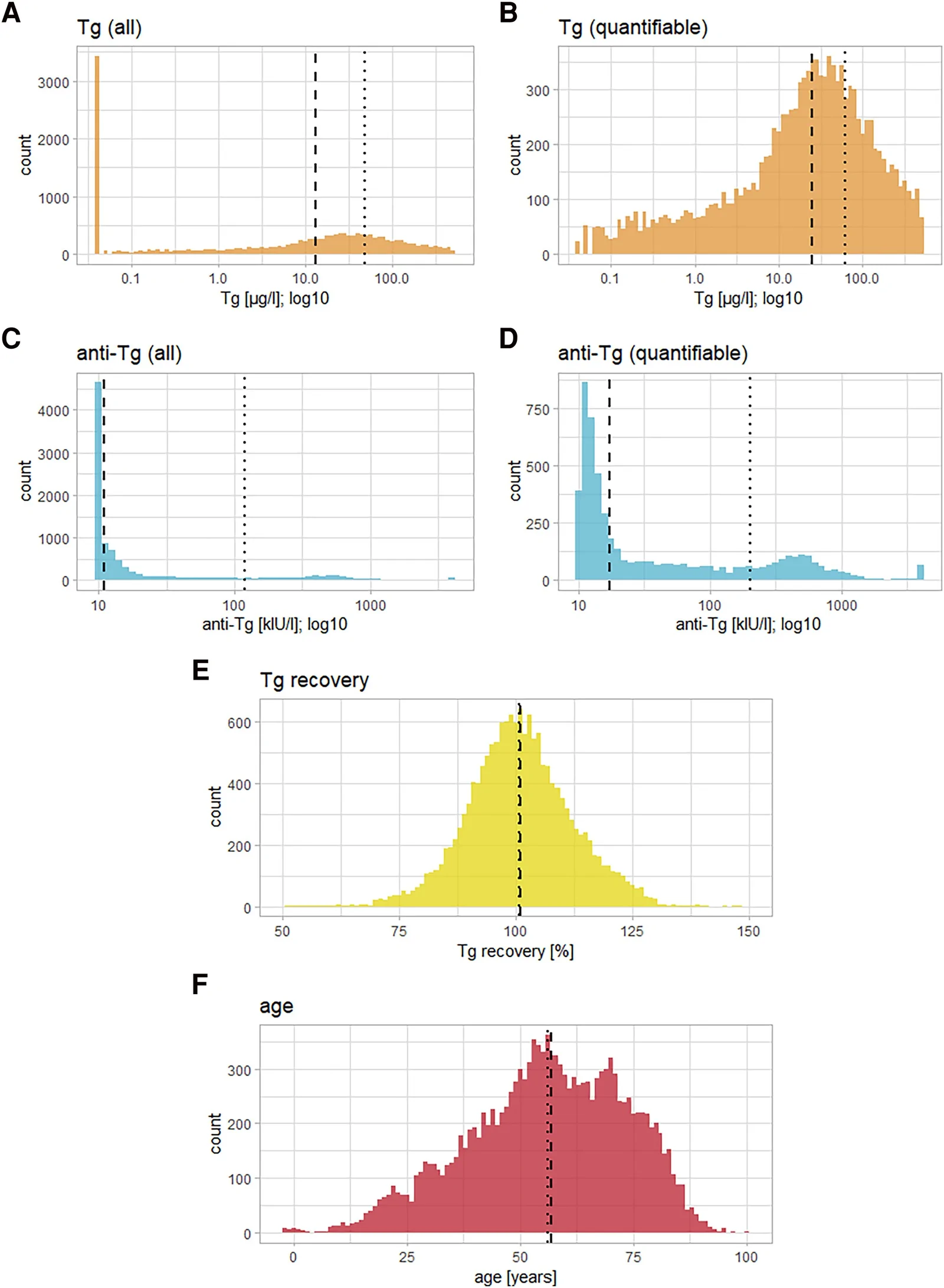
Distributions of measured levels of thyroglobulin (Tg) (A and B), anti-Tg (C and D), and Tg recovery (E) in all samples as well as age at sample collection (F). B and D only include values above the limit of quantification for Tg and anti-Tg, respectively. Dashed lines represent the respective median, dotted lines the mean. Note that A to D contain logarithmically scaled *x*-axes.

While the results for Tg recovery and patients’ ages were approximately normally distributed (Tg recovery range, 1-684%; median, 101 ± 10.4%; age range, 0-100 years; median: 57 ± 18.5 years), Tg approximated a log-normal distribution (range, <0.04-498 µg/L; median, 12.9 ± 19.1 µg/L), whereas anti-Tg approximated a Pareto distribution (range, <10->4000 kIU/L; median, 10.9 ± 1.48 kIU/mL). Both for Tg and anti-Tg, it was notable that a significant portion of samples returned results below the limit of quantification (Tg, 20.0%; anti-Tg, 43.3%). When these are disregarded, a second peak was notable for anti-Tg, which was an approximately log-normally distributed subset of samples with elevated levels of anti-Tg. See Fig. 1 for the distributions of all 3 variables. Using Spearman's rank correlation coefficient, no meaningful correlations between any of the continuous variables Tg, anti-Tg, Tg recovery, and age could be detected (ρ < |0.2| in all cases).

**Figure 2:**
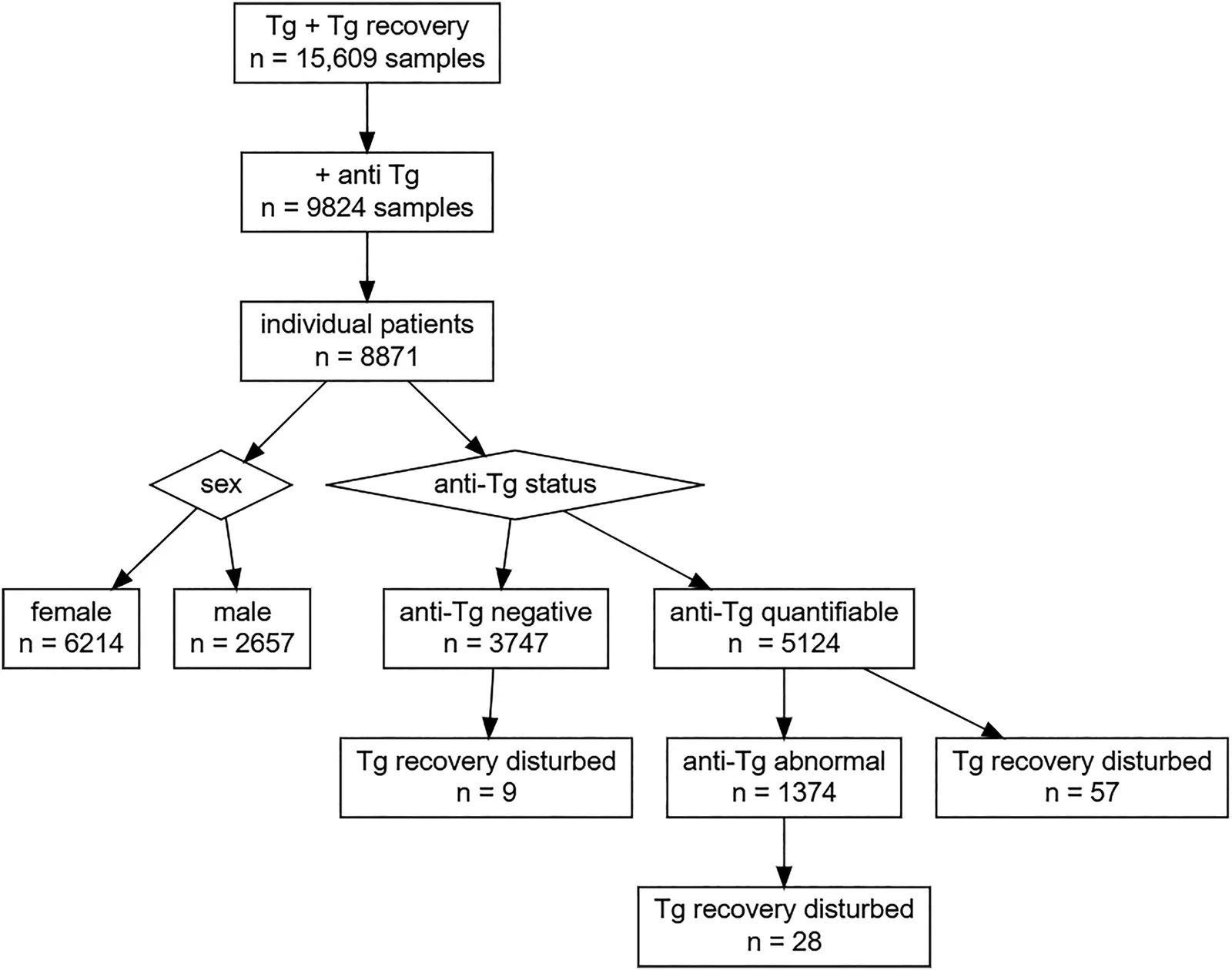
Flowchart of the different levels of subsets of samples and individual patients from whom samples were collected.

Out of all 14 338 patients from whom Tg was measured, only 83 (0.58%) exhibited a Tg recovery below the cutoff of 70% at any point. Of these 83, 11 patients with follow-up examinations also returned Tg recovery values within the reference range at different time points. Of the remaining 72 patients, who returned only abnormally decreased levels of Tg recovery, follow-up examinations were only available for 4 patients, the remaining 68 samples being isolated measurements of these patients. Of the 8871 patients for whom anti-Tg results were available, 1374 patients (15.5%) exhibited anti-Tg antibodies above the threshold of 115 kIU/L, and 5124 patients (57.8%) exhibited quantifiable levels of anti-Tg (above the threshold of 10 kIU/mL) at any point. Out of 66 samples (collected from 66 different patients) with a Tg recovery below the cutoff of 70%, for which anti-Tg was also measured, only 28 samples (42.4%) had an elevated level of anti-Tg, but 57 samples (86.4%) contained quantifiable levels of anti-Tg (see Fig. 2 for a flowchart of these different levels of subsets of samples and individual patients). χ² tests revealed that these distributions were significantly different from the rest of the cohort (*P* < .0001 for both elevated and quantifiable anti-Tg), although with a negligibly small effect size (Cramér's V, 0.06 and 0.04 for elevated and quantifiable anti-Tg, respectively). Of note, the lower limit of the reference range of Tg recovery of 70% is 2.8 SD below the mean of the Tg recovery in samples without quantifiable anti-Tg in our dataset. If the usual principles for the designation of reference ranges would be applied to Tg recovery in our dataset (which is ±1.96 SD from the mean of healthy subjects), a lower limit of 79.6% (approximately 80%) would result.

χ² tests revealed that both elevated levels and quantifiable levels of anti-Tg (measured in at least 1 sample) were significantly more common (albeit with small effect size) in women than in men (17.6% vs 10.4%; Cramér's V: 0.09; *P* < .0001 for elevated levels; 61.6% vs 48.7%; Cramér's V: 0.13; *P* < .0001 for quantifiable levels). Consequently, the same was true for levels of Tg recovery below 70% (0.84% vs 0.49%; Cramér's V: 0.11; *P* < .0001).

Via Mann-Whitney *U* test, we could identify statistically significant differences between the 2 sexes for anti-Tg (female vs male, 19.5 ± 13.3 kIU/mL vs 14.1 ± 5.19 kIU/mL; *P* < .0001; ρ = 0.15) and Tg recovery (99 ± 11.9 vs 100 ± 10.4; *P* < .0001; ρ = 0.07), albeit with small to very small margins of difference and effect sizes. We could not find such a difference between the sexes for Tg (*P* = .994).

Also via Mann-Whitney *U* test, we could show that samples with both elevated or only quantifiable levels of anti-Tg were collected on averagely younger patients than samples with normal or unquantifiable levels of anti-Tg (for elevated levels: 53.8 ± 21.0 vs 57.0 ± 18.0 years; *P* < .0001; ρ = 0.07; for quantifiable levels: 55.4 ± 19.0 vs 58.0 ± 17.8 years; *P* < .0001; ρ = 0.08). Somewhat in contrast to this, samples with Tg recovery below 70% were collected from averagely older patients than samples with Tg recovery ≥70% (62.3 ± 18.8 vs 56.6 ± 18.4 years; *P* = .004; ρ = 0.02). See Table 1 for a systematic overview of the comparisons performed as part of the descriptive statistics.

**Table 1:** Overview of statistical comparisons of the association between sex as well as age and patients’ status as regards levels of Tg, anti-Tg, and Tg recovery

Measure of comparison	Group	Value	Statistical test	Effect size	*P* value
Occurrence of elevated levels of anti-Tg	Female	17.6%	χ² test; Cramér's V for effect size	0.09	<.0001
	Male	10.4%			
Occurrence of quantifiable levels of anti-Tg	Female	61.6%	χ² test; Cramér's V for effect size	0.13	<.0001
	Male	48.7%			
Occurrence of disturbed Tg recovery [%]	Female	0.84%	χ² test; Cramér's V for effect size	0.11	<.0001
	Male	0.49%			
Level of anti-Tg, kIU/mL, (reference range <115 kIU/mL)	Female	19.5 ± 13.3	Mann-Whitney *U* test	ρ = 0.15	<.0001
	Male	14.1 ± 5.19			
Level of Tg recovery, % (Reference range, 70%-130%)	Female	99 ± 11.9	Mann-Whitney *U* test	ρ = 0.07	<.0001
	Male	100 ± 10.4			
Level of Tg, µg/L (reference range, 3.5-77 µg/L)	Female	24.6 ± 32.6	Mann-Whitney *U* test	N/A	.994
	Male	24.9 ± 33.2			
Age, years	Anti-Tg elevated	53.8 ± 21.0	Mann-Whitney *U* test	ρ = 0.07	<.0001
	Anti-Tg normal or unquantifiable	57.0 ± 18.0			
Age, years	Anti-Tg quantifiable, but not elevated	55.4 ± 19.0	Mann-Whitney *U* test	ρ = 0.08	<.0001
	Anti-Tg not quantifiable	58.0 ± 17.8			
Age, years	Tg recovery disturbed	62.3 ± 18.8	Mann-Whitney *U* test	ρ = 0.02	.004
	Tg recovery undisturbed	56.6 ± 18.4			

### Associations between Tg, anti-Tg, and Tg recovery

**Figure 3:**
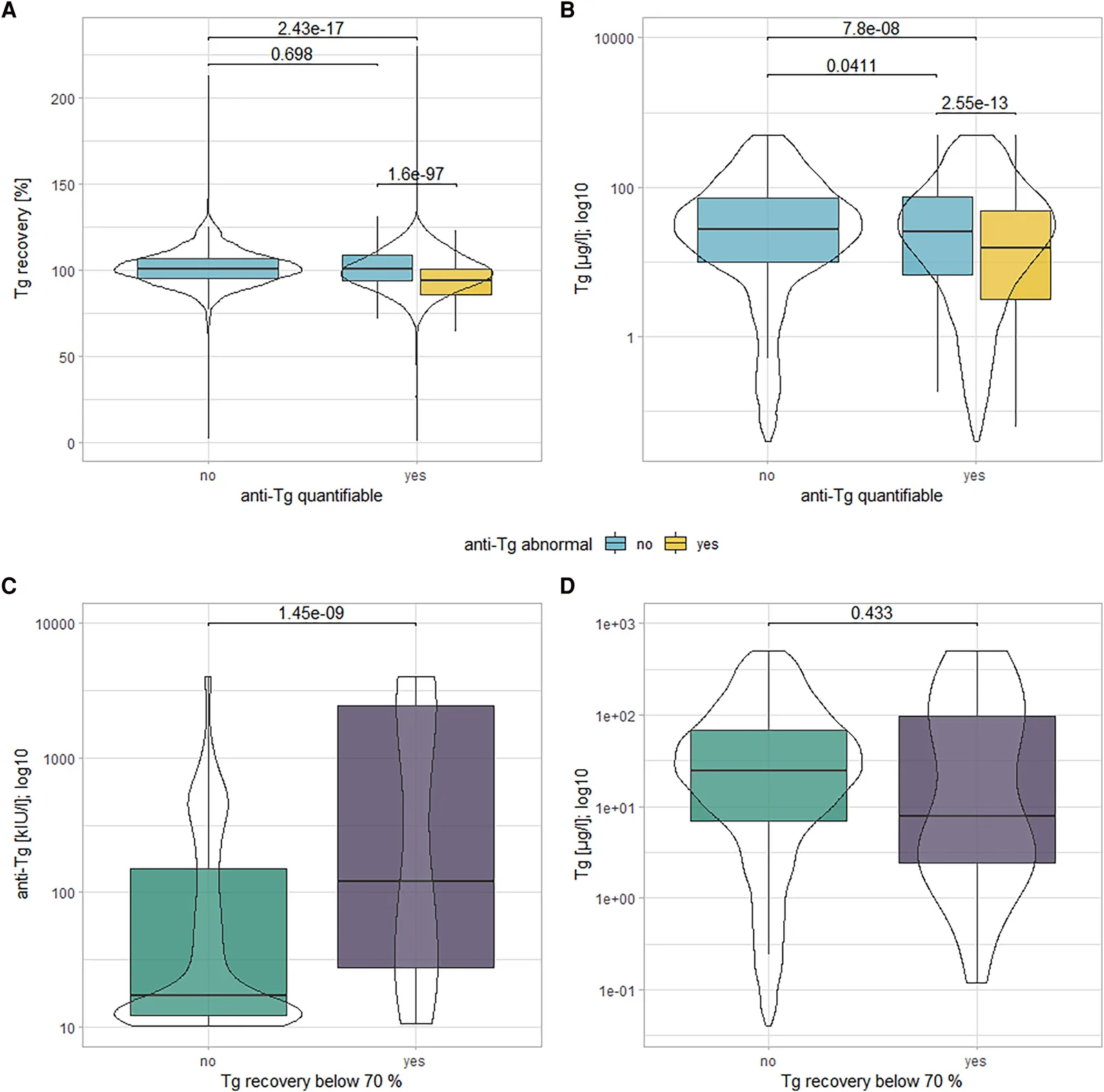
Levels of thyroglobulin (Tg) recovery (A) and Tg (B) depending on the anti-Tg status of the individual sample (anti-Tg not quantifiable, quantifiable but not elevated, or elevated) as well as levels of anti-Tg (C) and Tg (D) depending on the Tg recovery status of the sample (Tg recovery above or below the cutoff of 70%). Violin plots in A and B represent samples stratified only by quantifiability of anti-Tg, whereas boxplots are also stratified by level of quantifiable anti-Tg (elevated/not elevated).

The comparison between samples containing or not containing quantifiable levels of anti-Tg revealed statistically significantly lower levels of both Tg recovery and Tg itself for the former (Tg recovery: 99 ± 11.9% vs 101 ± 8.9%, *P* < .0001; ρ = 0.09; Tg: 21.7 ± 29.9 µg/L vs 26.6 ± 34.4 µg/L; *P* < .0001; ρ = 0.06) (Fig. 2A and 2B). Further analysis of the data showed, however, that this difference was mainly driven by the samples with elevated levels of anti-Tg. The comparison of samples with or without elevated levels of anti-Tg within the subgroup of samples with quantifiable levels of anti-Tg showed highly significant differences for both Tg-recovery and Tg (Tg recovery: 94 ± 10.4% vs 101 ± 10.4%; *P* < .0001; ρ = 0.281; Tg: 15.4 ± 21.3 µg/L vs 24.6 ± 33.7 µg/L; *P* < .0001; ρ = 0.11) (Fig. 3A and 3B), while the comparison of samples with or without quantifiable levels of anti-Tg within the subgroup of samples without elevated levels of anti-Tg showed that there was no statistically significant difference in Tg recovery (*P* = .698) (Fig. 3A) and a barely statistically significant difference for Tg (24.6 ± 33.7 µg/L vs 26.6 ± 34.4 µg/L; *P* = .041; ρ = 0.02) (Fig. 3B). Of note, the average Tg recovery of samples with elevated anti-Tg (94 ± 10.4%) was only 0.6 SD below the mean Tg recovery of samples without quantifiable anti-Tg, signifying a large overlap between both groups. The comparison between samples with a Tg recovery above and below the threshold of 70% showed significantly higher levels of anti-Tg for the latter group (17.1 ± 9.78 kIU/mL vs 103 ± 137 kIU/mL, *P* < .0001; ρ = 0.08) (Fig. 3C). For Tg, there was no such difference (*P* = .433) (Fig. 3D), although this comparison was likely underpowered.

**Figure 4:**
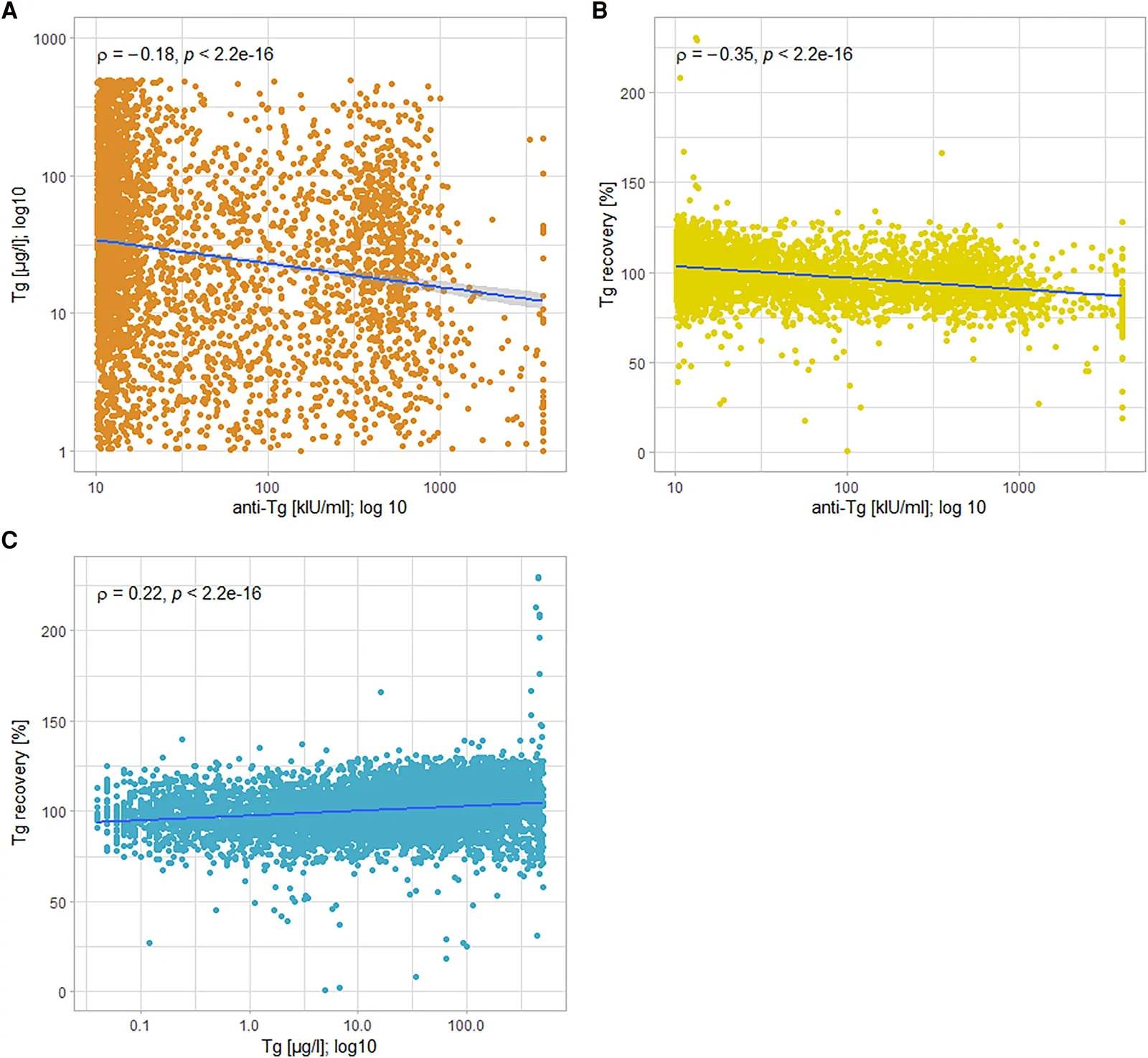
Correlations between thyroglobulin (Tg) and anti-Tg (A), Tg recovery and anti-Tg (B), and Tg recovery and Tg (C). Blue lines represent linear smoothed means, gray areas around them represent the 95% confidence interval. In the upper-left-hand corner of each panel are Spearman's ρ and the corresponding *P*-value. Axes are logarithmically scaled for Tg and anti-Tg.

Correlations of Tg, anti-Tg, and Tg recovery were statistically significant, albeit of only small to medium effect size. Both Tg (ρ = −0.18; *P* < .0001) (Fig. 4A) and Tg recovery (ρ = −0.35; *P* < .0001) (Fig. 4B) were correlated negatively with anti-Tg, while Tg recovery was correlated positively with Tg itself (ρ = 0.22; *P* < .0001) (Fig. 4C).

## Discussion

First, our results show that, within our cohort, levels of Tg recovery below the cutoff of 70% are a rarity (with only 0.58% of all samples measured), while elevated levels of anti-Tg are markedly more common (15.5%), and quantifiable levels of anti-Tg could even be detected in a majority of all samples (57.8%). Despite this discrepancy between the frequencies of pathologic results in Tg recovery and anti-Tg assays, we were able to detect a statistically significant impact of anti-Tg on both Tg recovery and the measurement of Tg itself. This impact is detectable both in lower levels of Tg and Tg recovery, especially in samples with elevated levels of anti-Tg, and in the negative correlation between Tg or Tg recovery and anti-Tg. It is worth noting, however, that even the median Tg recovery in samples containing elevated levels of anti-Tg is well within the reference range with 94% ± 10.4%, and effect sizes of these differences were very small. Furthermore, our findings show that, despite earlier findings that any level of anti-Tg can significantly interfere with Tg measurement (3, 4, 10), it is mainly elevated levels of anti-Tg that affect Tg recovery and the measurement of Tg itself. Samples with quantifiable, but not elevated levels of anti-Tg did not show significantly different levels of Tg recovery than did samples without quantifiable levels of anti-Tg. For Tg itself, this difference was marginal and barely reached statistical significance with a negligibly small effect size in an analysis of nearly 10 000 samples. Our results are comparable to those of Giovanella et al, who found that a so-called Tg mini-recovery assay (in which a test serum with only a very small concentration of 5 µg/L is added to the sample) was also rarely disturbed by anti-Tg in DTC patients; and when it was disturbed, it was by high levels of anti-Tg. In contrast to our findings, disturbances in Tg recovery testing were relatively frequent in the cohort of Giovanella et al, with 5% of all anti-Tg positive samples yielding a disturbed Tg mini-recovery (11). It is possible that this is a consequence of a more frequent interference of anti-Tg in Tg testing in DTC patients compared with other patient groups, but it might also be due to assay-specific effects. It should certainly caution us that results for 1 specific group of patients from one specific assay should not be generalized to other groups of patients or other assays.

In general, it can be stated that all statistically significant associations or differences detected in our study were of small to very small effect size. This is not to say that the detected differences are not real or completely insignificant. Rather, it must be stated that while anti-Tg certainly can interfere in the measurement of Tg recovery and of Tg itself, this interference appears to be of rather small effect—certainly too small, in any way, to be detected on a single-specimen basis by Tg recovery in its current form with its rather large reference range of 70% to 130% that leads to only the most extreme (and subsequently equally rare) cases of interference to be detected. While the lower limit of the reference range of 70% is therefore probably too low (lying as it does almost 3 SD below the mean Tg recovery of samples without quantifiable anti-Tg in our sample), even a more realistic limit of 80% (which is roughly 1.96 SD from the mean of samples without anti-Tg and therefore at the 2.5th percentile) would still be too broad for the average Tg recovery in samples with elevated anti-Tg, which, at 94% is a mere 0.6 SD below the mean of samples without anti-Tg. This leads us to conclude that Tg recovery in its current form and with its current reference range is not precise enough to reliably detect the interference of anti-Tg on the measurement of Tg (that does indeed occur, as our data confirm), at least not on the level of the individual specimen. Furthermore, while quantifiable and elevated results for anti-Tg are more common in samples with disturbed Tg recovery, far from all of those samples contain quantifiable or elevated levels of anti-Tg. This, in turn, makes us conclude that disturbed Tg recovery is not specific for the interference of anti-Tg in the measurement of Tg. Rather, the almost perfectly Gaussian distribution of Tg recovery values around a mean (and a median) close to 100% (with 95% of values being distributed within 2 SD of the mean) implies that at least some “disturbed” Tg recovery results are the consequence of mere stochastic inevitability in a set of normally distributed values. This is all the more relevant when considering that for the vast majority of patients with a disturbed Tg recovery in our cohort (68/83; 82%), this relied on the measurement of a single sample, making it nearly impossible to exclude that the disturbed Tg recovery was actually the consequence of 1 of many uncontrollable independent variables, such as measurement errors. Adding to this, Andress et al have shown that even in anti-Tg negative patients in which quantifiable levels of Tg could be detected in fine-needle aspiration biopsy washout fluid, serum Tg still remained negative, suggesting that anti-Tg is not the only explanation for falsely undetectable serum Tg (13).

Still, in the care of patients with DTC, especially in the follow-up of a thyroidectomy, it is especially very low amounts of Tg and thus also incremental changes in Tg levels that are of utmost importance, which is why even seemingly small interferences in the measurement of Tg cannot be disregarded as insignificant. However, the question is how to best identify those samples in which such an interference is present, as not all quantifiable levels of anti-Tg may interfere, while those that do may be present in small quantities. As previously detailed, the measurement of Tg recovery appears not to be ideally suited to identify meaningful interferences between anti-Tg and Tg. Barring the theoretical possibility of testing all samples both via immunometric assay and via another method that is less prone to interferences caused by anti-Tg, such as LC-MS/MS or radioimmunoassay, which is unrealistic, both because of the limited availability of LC-MS/MS and RIA and because many conventional LC-MS/MS and RIA assays are not sensitive enough to detect those very low levels of Tg that are of relevance in the follow-up treatment of DTC patients (8, 14), there is currently no accepted or standardized method to identify samples in which anti-Tg has interfered with the measurement of Tg with a reasonable degree of certainty. Most experts agree that anti-Tg should be assessed in every sample in which Tg is measured (14, 15) to detect possible interferences, but whether or not detected anti-Tg of any level really did cause an interference with Tg is at present probably best assessed by evaluating the clinical plausibility of the measured Tg levels. Furthermore, the consensus has generally shifted toward using anti-Tg as a surrogate tumor marker in those DTC patients in which they are present (instead of Tg itself), due to the discussed uncertainty of analytic interference of anti-Tg with the measurement of Tg. Giovanella et al were able to show that nondetectable levels of Tg are a reliable correlate of clinical remission of DTC in most patients, even those with anti-Tg, as anti-Tg very rarely drives Tg levels below the level of detection of an adequately sensitive assay, with only significantly increased levels of anti-Tg exerting a relevant influence on levels of Tg in isolated cases in their cohort (11). Our results support their conclusion that the level of anti-Tg and its development over time should be closely monitored, as elevated levels of anti-Tg apparently exerted a greater (albeit still small) effect on the measurement of Tg in our sample as well.

There are some additional findings from this study to discuss: both quantifiable and elevated levels of anti-Tg (and consequently, decreased levels of Tg recovery) were more common and the observed levels more pronounced in women than in men in our cohort. This finding is not surprising, as the female preponderance in autoimmune thyroiditis (AIT), of which anti-Tg is a marker, is well known (16). This, together with the fact that DTC is also more common in women (17), likely explains the roughly 2:1 ratio of female to male patients in our cohort. Considering that the female preponderance is even more pronounced in AIT than in DTC and that AIT is more common in younger individuals than DTC, it is also not surprising that male patients were, on average, somewhat older than female patients. This explanation is supported by the fact that patients with both elevated or quantifiable levels of anti-Tg were significantly younger than their respective counterparts. Also, there was a significant correlation of small effect size between Tg and Tg recovery. This suggests that Tg recovery is marginally greater when Tg levels within the sample are greater. While statistically significant, this finding is of virtually no clinical relevance, as the mean Tg recovery at every level of Tg is close to 100% and therefore well within the reference range.

Our study has several limitations: As samples were included in our analyses very broadly as part of our big data approach, the underlying study population has to be assumed to hail from a probably very heterogeneous population who presented at the hospital; the prevalences and demographic distributions we found cannot be generalized for any specific group of patients, be it AIT or DTC patients. Adding to this, we had no access to clinical data connected to the samples (such as exact diagnoses or possible treatment with thyroid hormones), further restricting the possibility of drawing conclusions specific to any clinical subgroup. Furthermore, as mentioned previously, the comparisons of samples with a Tg recovery above or below 70% was likely underpowered, as only 98 samples out of more than 15 000 showed a Tg recovery below 70%.

In conclusion, our data support the claim that Tg recovery testing is not sufficient to reliably detect interferences of anti-Tg in the measurement of Tg on a single-specimen level. While effects of anti-Tg on the measured levels of Tg recovery and of Tg itself could be detected, these were of small to negligible effect size. Much research is yet to be done to find ways of assessing low levels of Tg without the possibility of interference by anti-Tg or to find ways of reliably detecting this interference when it occurs.

## Data Availability

All datasets generated and analyzed during the current study are not publicly available but are available from the corresponding author on reasonable request.
